# Dogs as New Hosts for the Emerging Zoonotic Pathogen *Anaplasma capra* in China

**DOI:** 10.3389/fcimb.2019.00394

**Published:** 2019-11-26

**Authors:** Ke Shi, Junqiang Li, Yaqun Yan, Qian Chen, Kunlun Wang, Yongchun Zhou, Dongfang Li, Yuancai Chen, Fuchang Yu, Yongshuai Peng, Longxian Zhang, Changshen Ning

**Affiliations:** ^1^College of Animal Science and Veterinary Medicine, Henan Agricultural University, Zhengzhou, China; ^2^International Joint Research Laboratory for Zoonotic Diseases of Henan, Zhengzhou, China; ^3^Scientific Research Experiment Center & Laboratory Animal Center, Henan University of Chinese Medicine, Zhengzhou, China

**Keywords:** *Anaplasma capra*, dogs, hosts, tick-borne, zoonotic

## Abstract

*Anaplasma capra* is an emerging zoonotic tick-borne pathogen with a broad host range, including many mammals. Dogs have close physical interactions with humans and regular contact with the external environment. Moreover, they have been previously reported to be hosts of *Anaplasma phagocytophilum, A. platys, A. ovis*, and *A. bovis*. To confirm whether dogs are also hosts of *A. capra*, pathogen DNA was extracted from blood samples of 521 dogs, followed by PCR amplification of the citrate synthase (*gltA*) gene, heat shock protein (*groEL*) gene, and major surface protein 4 (*msp4*) gene of the *A. capra*. A total of 12.1% (63/521) of blood samples were shown to be *A. capra*-positive by PCR screening. No significant differences were observed between genders (*P* = 0.578) or types (*P* = 0.154) of dogs with *A. capra* infections. However, significantly higher *A. capra* infections occurred in dogs with regular contact with vegetation (*P* = 0.002), those aged over 10 years (*P* = 0.040), and during the summer season (*P* = 0.006). Phylogenetic analysis based on *gltA, groEL*, and *msp4* sequences demonstrated that the isolates obtained in this study were clustered within the *A. capra* clade, and were distinct from other *Anaplasma* species. In conclusion, dogs were shown to be a host of the human pathogenic *A. capra*. Considering the affinity between dogs and humans and the zoonotic tick-borne nature of *A. capra*, dogs should be carefully monitored for the presence of *A. capra*.

## Introduction

Vector-borne diseases are major causes of morbidity and mortality in dogs and are potentially of great public health importance because of their zoonotic nature and the role of pets as reservoirs (Xu et al., 2015; Cui et al., 2017; Maggi and Krämer, 2019). In the last few years, the number and range of species kept as companion animals have risen, and they are maintaining increasingly close interactions with humans in industrialized societies (Cito et al., 2016). Although the phenomenon of all companion animals, especially dogs and cats, sharing the same environment as humans is long-standing (Fang et al., 2015; Cui et al., 2017), urbanization has affected the emergence and increasing incidence of tick-borne diseases (TBD) (Fang et al., 2015). Together, these changes in human activity and the increased contact between humans, their pets, and nature have contributed to the rising abundance of tick exposure (Uspensky, 2014; Fang et al., 2015). Dogs are particularly unusual companion animals because of their outdoor nature and close relationship with humans. They can therefore act as good sentinels for human tick-borne infections, suggesting that their role as hosts of ticks and tick-borne pathogens should be monitored (Hornok et al., 2013; Vlahakis et al., 2018).

*Anaplasma* species are zoonotic pathogens with tick vectors and mammalian reservoir hosts (Li et al., 2015). To date, three *Anaplasma* species have been identified as human pathogens: *A. phagocytophilum, A. ovis*, and *A. capra* (Chen et al., 1994; Chochlakis et al., 2010; Li et al., 2015). *A. phagocytophilum* was first confirmed as the causative agent of human granulocytic anaplasmosis (HGA) in the USA in 1994 (Chen et al., 1994). In China, the first suspected human case was described in Anhui in 2006 (Zhang et al., 2008). The severity of HGA ranges from an asymptomatic infection to a mild or severe febrile illness with multiple organ failure or even death (Li et al., 2015). Besides humans, hosts of *A. phagocytophilum* include domestic and wild animals such as cattle, sheep, goats, horses, dogs, hares, yaks, and rodents (Fang et al., 2015). An *A. ovis* variant was first identified in a patient with fever, hepatosplenomegaly, and lymphadenopathy in Cyprus in 2007 (Chochlakis et al., 2010). Hosts of this pathogen include domestic goats, sheep, deer, wild boar, and domestic dogs (Yabsley et al., 2005; Aktas et al., 2010; Pereira et al., 2016; Cui et al., 2017; Wei et al., 2017). *A. capra* is a novel *Anaplasma* species so-called because it was originally found in asymptomatic goats; soon after, it was identified in 28 patients in Heilongjiang, China (Beyer and Carlyon, 2015; Li et al., 2015; Yang et al., 2018). Clinical features of *A. capra* infection in humans include the acute onset of fever, headache, and malaise, but it is very difficult to distinguish from other acute febrile illnesses, thus leading to misdiagnosis (Li et al., 2015). Subsequent reports have shown that *A. capra* is widely distributed throughout China (Yang et al., 2018). It appears to have a broad host range and genetic diversity, with other mammalian hosts including goats, sheep, cattle, deer, serows, takins, and Reeves' muntjacs worldwide (Sato et al., 2009; Sun et al., 2015; Yang et al., 2017, 2018; Peng et al., 2018; Seo et al., 2018).

As one of the main mammalian hosts of *Anaplasma* species, dogs have been shown to carry *A. phagocytophilum, A. platys, A. ovis*, and *A. bovis* pathogens in recent years in China (Zhang et al., 2012; Li et al., 2014; Cui et al., 2017). However, there are no reports about dogs as hosts of *A. capra* worldwide. To provide further information about the host range, clinical symptoms, and risk factors of *A. capra* infections, the detection of this pathogen was carried out in dogs in Henan Province, China.

## Materials and Methods

### Sample Collection

During 2013–2018, blood sample collection from dogs was carried out at six sampling sites in Zhengzhou city, Henan Province, central China. These included three pet hospitals (Pet clinic 1, Pet clinic 2, and Pet clinic 3) and three rescue centers for stray dogs (Stray dog rescue center 1, Stray dog rescue center 2, and Stray dog rescue center 3). Blood samples from pet clinics were obtained during outpatient testing, and detailed medical records were also collected. Blood samples from stray dog rescue centers were collected from a random proportion (5–10%) of dogs with the assistance of an experienced veterinarian. All blood samples were collected from the anterior tibial vein of the dogs with the help of a pet doctor or local veterinarian.

A total of 521 EDTA-K2 whole blood samples from different types of dogs were collected. Information about pet dogs, including age, gender, and clinical features, was obtained from sampling records, descriptions of chief complaints, and veterinarian diagnoses. However, few documents about stray dogs were available, and a review of their clinical symptoms suggested that they were healthy.

### DNA Extraction

Pathogen genomic DNA was extracted from 200 μL blood samples using a Blood DNA Kit (Omega Biotek Inc., Norcross, GA, USA) according to the manufacturer's protocol. The quantity and quality of the extracted DNA were evaluated using a NanoDrop™ 2000 spectrophotometer (Thermo Fisher Scientific, Waltham, MA), and it was stored at −20°C before use.

### PCR Amplification

DNA samples were screened for the presence of *A. capra* by PCR amplification of the citrate synthase gene (*gltA*), heat shock protein gene (*groEL*), and major surface protein 4 gene (*msp4*) using previously described primers and PCR conditions (Table 1). Each DNA sample was screened for all three *A. capra* genes, and the successful amplification of any one of the three genes was taken to indicate positive infection. Each PCR reaction was conducted at least twice using nuclease-free water as a negative control, and DNA extracted from sheep infected with *A. capra* (GenBank accession nos. MG879297, MH174929, and MH174932) was used as a positive control. PCR reactions were performed in an ABI 2720 thermal cycler (Life Technologies Holdings Pte Ltd., Singapore). The products were examined by agarose gel electrophoresis and visualized after staining with GelRed (Biotium Inc., Hayward, CA).

**Table 1:** Primers and PCR amplification conditions of *A. capra*.

**Target gene**	**Primer name**	**Primer sequence (5^**′**^−3^**′**^)**	**Annealing temperature**	**Amplicon size**	**References**
*glt A*	Outer-f	GCGATTTTAGAGTGYGGAGATTG	55°C	1031 bp	Li et al., 2015
	Outer-r	TACAATACCGGAGTAAAAGTCAA			
	Inner-f	TCATCTCCTGTTGCACGGTGCCC	60°C	594 bp	Yang et al., 2017
	Inner-r	CTCTGAATGAACATGCCCACCCT			
*groEL*	Forward	TGAAGAGCATCAAACCCGAAG	55°C	874 bp	Yang et al., 2017
	Reverse	CTGCTCGTGATGCTATCGG			
*msp4*	Forward	GGGTTCTGATATGGCATCTTC	53°C	656 bp	
	Reverse	GGGAAATGTCCTTATAGGATTCG			

### Sequencing and Phylogenetic Analysis

Positive PCR products were purified using Montage PCR filters (Millipore, Bedford, MA) and sequenced using a BigDye Terminator v 3.1 cycle sequencing kit (Applied Biosystems, Foster City, CA) on an ABI 3730 DNA analyzer (Applied Biosystems). Nucleotide sequences were confirmed by bidirectional sequencing and by sequencing a new PCR product if necessary. They were then compared with reference sequences downloaded from the National Center for Biotechnology Information (https://www.ncbi.nlm.nih.gov/) using ClustalX 2.1 (http://www.clustal.org/) to determine new variant strains of *A. capra*.

Phylogenetic trees were conducted by Bayesian inference (BI) and Monte Carlo Markov Chain methods in MrBayes v 3.2.6 (http://mrbayes.sourceforge.net/). FigTree v 1.4.4 (http://tree.bio.ed.ac.uk/software/figtree/) was used to visualize and edit the maximum clade credibility tree generated by these analyses. Posterior probability values were estimated based on 1,000,000 generations with four simultaneous tree building chains, with trees being saved every 100th generation. A 50% majority rule consensus tree for each analysis was constructed based on the final 75% of trees generated by BI.

Sequences similarity were further analyzed by DNAStar Laser-gene program (DNAStar Inc., Madison, WI, USA) to evaluated the homology of the sequences obtained in the present with the sequences downloaded from the National Center for Biotechnology Information (https://www.ncbi.nlm.nih.gov/) (Figure S2).

### Statistical Analysis

Variations in *A. capra* infection of dogs at different locations, seasons, age groups, genders, clinical signs, and use of vermifuge were analyzed by Fisher's exact test with SPSS (version 22.0) software. Differences were considered statistically significant if *P* < 0.05. Odds ratios (ORs) and their 95% confidence intervals (95% CIs) were estimated to explore the strength of the association between *A. capra*-positivity and the conditions tested. Then, the significant different correlation factors were used to process the canonical correspondence analysis by canoco5 (http://www.canoco5.com) (Figure S1).

### Nucleotide Sequence Accession Numbers

The representative sequences obtained in this study have been submitted and deposited in the GenBank database with the following accession numbers: *gltA* (MK838608 and MK838609), *groEL* (MK862099), and *msp4* (MK838605– MK838607).

### Ethics Statement

This study was carried out in accordance with the Chinese Laboratory Animal Administration Act (1988) after it was reviewed and its protocol was approved by the Research Ethics Committee of Henan Agricultural University. Appropriate permission was gained from the dog owners before the collection of blood specimens.

## Results

### Infection of Dogs With *A. capra*

A total of 63 of the 521 dog blood samples were *A. capra*-positive by PCR screening (12.1%, 95% CI: 9.3–14.9). The infection rates of *A. capra* in pet dogs and stray dogs were 12.9% (59/458, 95% CI: 9.8–16.0) and 6.3% (4/63, 95% CI: 0.2–12.5), respectively, There were no significant differences in the infection ratio of *A. capra* in pet dogs and stray dogs (*P* = 0.154) (Table 2).

**Table 2:** Univariable and multivariable analyses of risk factors associated with *A. capra* in dogs.

**Variables**	**No. tested**	**No. positive**	**Infection rate (%) 95% CI**	**OR 95% CI**	***P***
**Sampling sites^a^**					
Pet clinic 1	233	43	18.5 (13.4–23.5)	1	0.002
Pet clinic 2	77	3	3.9 (0.0–8.3)	0.179 (0.054–0.595)	0.001
Pet clinic 3	148	13	8.8 (4.2–13.4)	0.425 (0.220–0.822)	0.006
Stray dog rescue center1	20	3	15.0 (0.0–32.1)	0.780 (0.219–2.780)	1.000
Stray dog rescue center2	24	1	4.2 (0.0–12.8)	0.192 (0.025–1.462)	0.090
Stray dog rescue center3	19	0	0.0		
**Kinds of dogs^a^**					
Pet dogs	458	59	12.9 (9.8–16.0)	1	
Dogs in Stray dog rescue center	63	4	6.3 (0.2–12.5)	0.458 (0.161–1.309)	0.154
**Seasons^a^**					
Spring (Mar.-May)	354	33	9.3 (6.3–12.4)	0.401 (0.233–0.689)	0.001
Summer (Jun. –Aug.)	142	29	20.4 (13.7–27.1)	1	0.006
Autumn (Sep. –Nov.)	10	0	0.0		
Winter (Dec. –Feb.)	15	1	6.7(0–21.0)	0.278 (0.035–2.204)	0.306
**Age groups^b^**					
>10 year	15	5	33.3 (6.3–60.4)	1	0.040
5–10 year	40	7	17.5 (5.2–29.8)	0.424 (0.110–1.634)	0.274
1–5 year	147	20	13.6 (8.0–19.2)	0.315 (0.098–1.017)	0.059
6–12 month	92	9	9.8 (3.6–16.0)	0.217 (0.061–0.776)	0.026
3–6 month	93	6	6.5 (1.4–11.5)	0.138 (0.036–0.535)	0.007
<3 month	71	12	16.9 (8.0–25.8)	0.407 (0.118–1.406)	0.164
Unknown	63	4	6.3 (0.2–12.5)		
**Gender^b^**					
Female	202	28	13.9 (9.1–18.7)	1	
Male	256	31	12.1 (8.1–16.1)	0.856 (0.495–1.481)	0.578
Unknown	63	4	6.3 (0.2–12.5)		
**Influenza**–**like illness symptoms^c^**					
Yes	312	37	11.9(8.3–15.5)	1	
No	146	22	15.1(9.2–20.9)	1.319 (0.747–2.329)	0.370
Unknown	63	4	6.3 (0.2–12.5)		
**Anemia symptoms or not^c^**					
Yes	18	6	33.3(9.2–57.5)	1	
No	440	53	12.0(9.0–15.1)	0.274 (0.099–0.760)	0.019
Unknown	63	4	6.3 (0.2–12.5)		
**Vermifuge used or not^b^**					
Yes	136	20	14.7 (8.7–20.7)	1	
No	322	39	12.1 (8.5–15.7)	0.854 (0.479–1.524)	0.651
Unknown	63	4	6.3 (0.2–12.5)		
Total	521	63	12.1 (9.3–14.9)		

a *Sampling record*.

b *Chief complaint*.

c *Veterinarian diagnosis*.

### Distribution of *A. capra* Infections

*A. capra* infections in dogs with different types of samples were analyzed (Table 2). *A. capra* infections were found in five of the six sampling sites, at rates ranging from 0–18.5%, with significant differences among sites (*P* = 0.002). Additionally, higher infection rates were observed in dogs older than 10 years (33.3%, 95% CI: 6.3–60.4), and lower rates were seen in dogs aged 3–6 months compared with other age groups (6.5%, 95% CI: 1.4–11.5) (*P* = 0.040). As expected, the highest infections were documented in the summer months (20.4%, 95% CI: 13.7–27.1) (*P* = 0.006), and in dogs with anemic symptoms such as pale mucous membranes (*P* = 0.019). However, there were no differences in infection rates between dogs of different genders (*P* = 0.578), nor in those with influenza-like illness symptoms including fever, cough, malaise, and depression (*P* = 0.370), nor in those administered vermifuge in the last month (*P* = 0.651).

### Sequences and Phylogenetic Analysis

**Figure 1:**
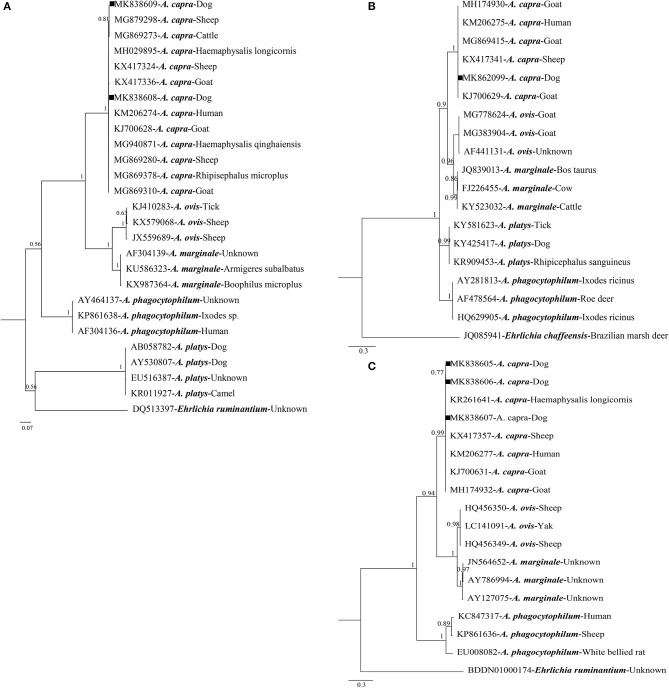
Bayesian phylogenetic analysis of *A. capra* based on *gltA* **(A)**, *groEL* **(B)**, and *msp4* **(C)** sequences. Significant posterior probabilities are indicated at branches. Sample names include GenBank accession numbers followed by *Anaplasma* spp. *Ehrlichia ruminantium* and *Ehrlichia chaffeensis* were used as outgroups. The sequences identified in this study are marked by squares.

Of the 63 *A. capra*-positive specimens, 26 were PCR-positive based on *gltA*, 17 based on *groEL*, and 61 based on *msp4*. Sequence analysis showed that *gltA* sequences were divided into two distinct sequence types: one (including 20 isolate sequences, MK838608) shared 100% identity with *A. capra* isolates from China, with previous hosts including humans (KM206274), goats (KM869310), sheep (KM869280), and ticks (MG940871 and MH029895). The others (including six isolates sequences, MK838609) showed 99.7% identity with *A. capra* isolates from humans (KM206274) (Figure S2A). Phylogenetic analysis based on *gltA* sequences revealed that the two types were in the same *Anaplasma* species clade (Figure 1). These isolates were closely related to *A. capra*, but distinct from other known *Anaplasma* species (Figure 1).

Sequence analysis revealed that *groEL* gene sequences obtained in this study (MK862099) were 100% identical to each other. Findings showed that the sequence had two single nucleotide polymorphisms (SNPs; G→ A and T→ A substitutions at positions 445 and 827, respectively) compared with the human isolate sequence (KM206275), and shared 99.9% identity with sequences isolated from sheep (KX417341), goats (KJ700629), and ticks (KX987393) (Figure S2B). Phylogenetic analysis based on *groEL* gene sequences demonstrated that the isolates were clustered within the *A. capra* clade, but distinct from other well-defined *Anaplasma* species (Figure 1).

For *msp4* gene sequences, we acquired three different sequences (MK838605–MK838607) from dogs in the present study. One (KM838607) shared 100% identity with those of *A. capra* isolates from humans (KM206277), and the others had one (T→ C at position 320) or two (C→ T and T→ C substitutions at positions 246 and 320, respectively) SNPs compared with the human sequence (KM206277), and shared 99.7–99.8% similarity with sequences of *Anaplasma* spp. from humans (KM206277), goats (KJ700631), sheep (KX417357), and ticks (KR261641) (Figure S2C). Phylogenetic analysis based on *msp4* sequences demonstrated that the isolates were clustered within the *A. capra* clade, but distinct from other well-defined *Anaplasma* species (Figure 1).

## Discussion

The natural infection cycle of *Anaplasma* species is dependent upon the presence of tick vectors and infected vertebrate reservoir hosts (de la Fuente et al., 2016). *Anaplasma* species are transmitted by ixodid ticks transstadially rather than transovarially, so reservoir hosts play a crucial role in the maintenance and spread of these pathogens (Yang et al., 2018). Currently, a number of vertebrate animals are considered competent hosts for *Anaplasma*, including humans, cattle, sheep, goats, dogs, horses, and deer (Stuen et al., 2013). Moreover, dogs have been reported as hosts of *A. phagocytophilum, A. platys, A. ovis*, and *A. bovis* (Zhang et al., 2012; Li et al., 2014; Cui et al., 2017).

Host and environmental factors are thought to play an important role in the epidemiology of TBD in dogs (Abd Rani et al., 2011). Furthermore, high-quality vegetative cover may have increased the abundance and diversity of ticks (Fang et al., 2015). A previous study showed that TBD were less likely to infect dogs from refuges and animals fed nutritious diets, even after adjusting for the presence of ticks, than free-roaming strays (Abd Rani et al., 2011). However, we found no difference in the natural infection rate of *A. capra* between pet and stray dogs (*P* = 0.154).

The novel tick-transmitted zoonotic *A. capra* was identified in dogs from five of six sampling sites in the present study, with a total infection rate of 12.1% (63/521). Likewise, *A. ovis, A. bovis*, and *A. phagocytophilum* have been identified in dogs in Henan with prevalences of 6.2%, 4.1%, and 0.4%, respectively (Cui et al., 2017), suggesting that *Anaplasma* species are common pathogens of dogs in Henan. We detected the highest infection rate in Pet clinic 1 (*P* = 0.002), which is located in a suburban area of the city surrounded by rich vegetation. Similar findings were previously observed for the tick-borne fever with thrombocytopenia syndrome which was also significantly associated with vegetation-rich regions (Liu et al., 2014; Fang et al., 2015).

We observed the highest *A. capra* infection rate in dogs older than 10 years of age. Similarly, other canine vector-borne disease pathogens such as *A. phagocytophilum* and *B. burgdorferi* were detected at higher infection rates in Korean dogs aged over 2 years (Lim et al., 2010), while no significant difference in *A. platys* infection was observed among various groups of dogs (Kamani et al., 2013). Dogs aged over 10 years, so-called elderly dogs, may have a poor physical condition and be more easily infected by pathogens. Furthermore, *A. capra* infection may be chronic and persistent infection, so the potential role of elderly dogs as carriers of *A. capra* should be noted. We observed no difference in infection rate between dogs of different genders, which is consistent with previous reports about tick-borne pathogens (Lim et al., 2010; Hornok et al., 2013; Peng et al., 2018).

Our study also revealed a high prevalence of *A. capra* infection during the summer season. Similar results were reported for *Anaplasma* species such as *A. bovis, A. ovis*, and *A. capra* in sheep, goats, and cattle (Belkahia et al., 2017; Seo et al., 2018). All *Anaplasma* species are transmitted to their natural hosts by ticks, and the warm summer season offers favorable conditions for tick distribution (Belkahia et al., 2017). Thus far, *A. capra* has also been detected in ticks such as *Ixodes persulcatus, Haemaphysalis longicornis*, and *Haemaphysalis qinghaiensis* in many places in China (Peng et al., 2018). From spring to autumn, ticks are found throughout China and demonstrate extended periods of activity; therefore with rising tick numbers, the risks of host infection with tick-borne pathogens also increase (Chvostáč et al., 2018; Jaimes-Dueñez et al., 2018).

Clinical features of human infection by *A. capra* include an influenza-like illness such as fever, headache, malaise, and chills (Li et al., 2015). In the present study, we found no difference in *A. capra* infections between dogs with and without influenza-like illness symptoms. However, a significant difference was detected between dogs with and without symptoms of anemia such as pale mucous membranes. However, Li and colleagues (Li et al., 2015) did not describe morulae or other forms of *A. capra* in peripheral blood smears, but instead found that *A capra* was more closely related to species that infect mammalian erythrocytes; thus, they anticipated intracellular *A capra* infection in mammalian erythrocytes. The correlation of anemia with *A. capra* infection should nonetheless be verified in a future study, while the present study describes the clinical features of *A. capra* infection in dogs for the first time.

Phylogenetic analysis of *A. capra* based on *gltA, groEL*, and *msp4* showed that the isolate sequences obtained in the present study, as well as those previously isolated from sheep, ticks, goats, and humans, formed an independent clade clearly distinct from other members of *Anaplasma* species (Li et al., 2015; Yang et al., 2017; Guo et al., 2018; Peng et al., 2018; Figure 1). For *gltA*, one sequence (MK838608) obtained in this study shared 100% identity with an *A. capra* isolate (KM206274) from humans, while the other sequence (MK838609) shared 99.7% identity with the same isolates from humans (Li et al., 2015). For *groEL*, the sequence acquired from dogs (MK862099) shared 99.9% identity with the sequence from humans (KM206275), and three different *msp4* sequences obtained in the present study shared 99.7–100% similarity with sequences of *Anaplasma* species from humans (KM206277) (Li et al., 2015). Phylogenetic analysis indicated that *A. capra* identified in this study was highly similar to sequences obtained from humans (KM206274, KM206275, and KM206277), in view of the affinity between dogs and humans, indicating that a high level of attention should be paid to *A. capra* infection in dogs for public health reasons.

A large proportion of *A. capra* sequences (20/26) obtained from dogs in this study showed 100% identity with isolates from ticks, sheep, goats, and humans (Li et al., 2015; Yang et al., 2017; Guo et al., 2018; Peng et al., 2018). Furthermore, many domestic animals like dogs may serve as a suitable reservoir or a dead end host of *Anaplasma* species in urban areas (Schorn et al., 2011). Companion animals are becoming increasingly popular in China, and the transport of dogs to other areas adds to the spread of pathogens (Stuen et al., 2013). Considering dogs as hosts both of *Anaplasma* species and ticks, together with the growing affinity between humans and dogs, we should not ignore their important role in spreading *A. capra* infection in areas where tick vectors are abundant.

## Conclusion

The present study documents dogs as a new host for *A. capra* for the first time. It is noteworthy that older dogs are more readily infected by *A. capra*. The risk factor for infection is predicted to increase with rising numbers of ticks in hot seasons and with increasing contact between dogs and ticks. Hence, we advise that dog owners prevent their pets from entering woods, copses, mountainsides, and grass close to rivers during peak tick seasons to limit vector contact. Further research should also evaluate whether dogs are competent reservoirs of *A. capra*.

## Data Availability Statement

The datasets generated for this study can be found in the GenBank database with the following accession numbers: gltA (MK838608 and MK838609), groEL (MK862099), and msp4 (MK838605–MK838607).

## Ethics Statement

The animal study was reviewed and approved by Chinese Laboratory Animal Administration Act (1988); Research Ethics Committee of Henan Agricultural University. Written informed consent was obtained from the owners for the participation of their animals in this study.

## Author Contributions

CN conceived the study. CN and LZ designed the experiments. KS, YY, QC, KW, YZ, and YP performed the experiments. KS, DL, YC, and FY performed data analysis. KS, JL, and CN wrote the manuscript. All authors approved the final version of the manuscript.

### Conflict of Interest

The authors declare that the research was conducted in the absence of any commercial or financial relationships that could be construed as a potential conflict of interest.

## Supplementary Material

Figure S1 Results of Canonical correspondence Analysis (CCA) between the prevalence of *A. capra* and several variables.

Figure S2 The homology analysis of *A. capra* based on *gltA* **(A)**, *groEL* **(B)**, and *msp4* **(C)** sequences.
