# Supplementary figures and images for: Dogs as New Hosts for the Emerging Zoonotic Pathogen Anaplasma capra in China
Source: Front Cell Infect Microbiol. 2019 Nov 26;9:394. doi: 10.3389/fcimb.2019.00394 (PMC6901931; doi:10.3389/fcimb.2019.00394)

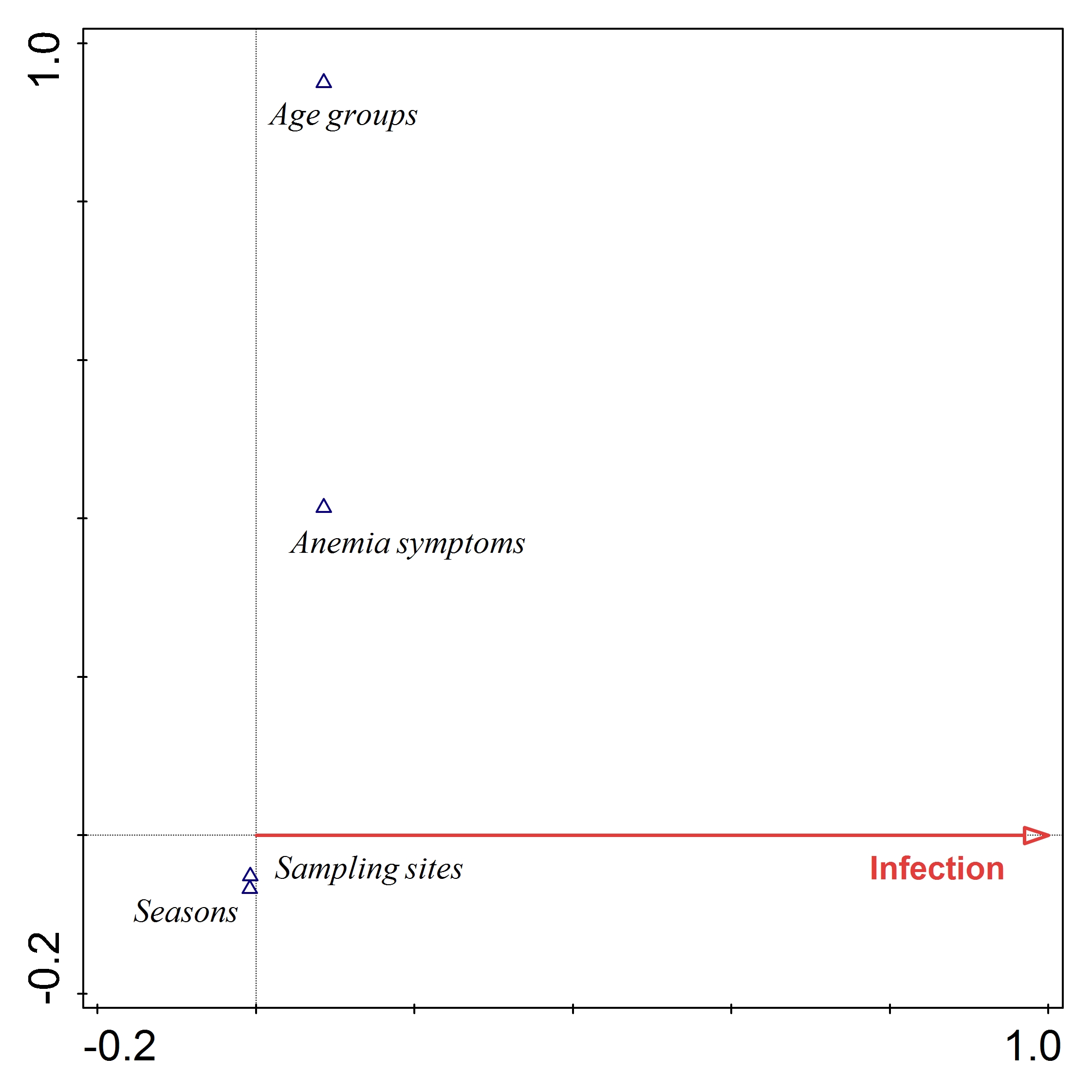

Supplement: Figure S1 — Results of Canonical correspondence Analysis (CCA) between the prevalence of A. capra and several variables. [file Image_1.JPEG]

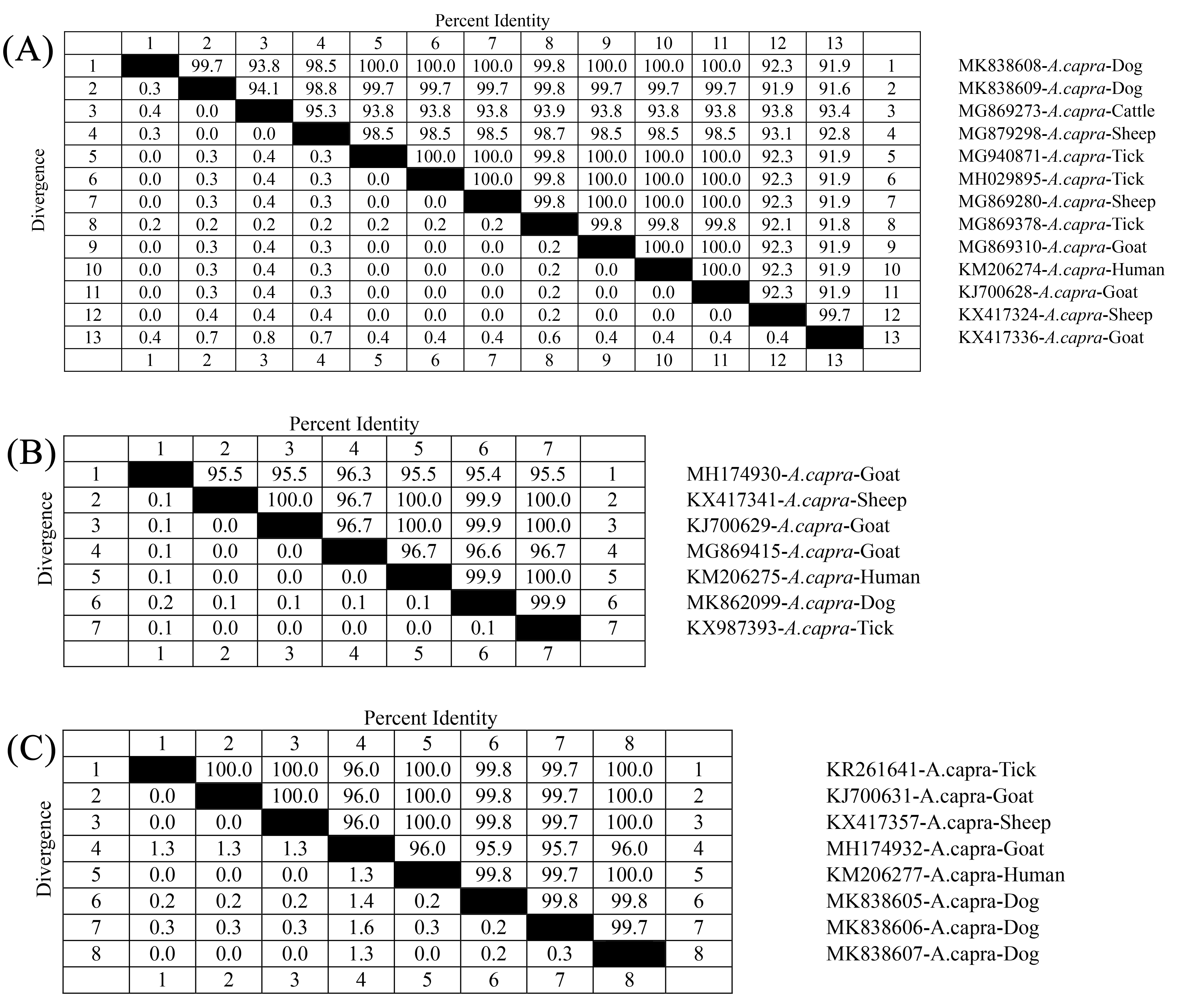

Supplement: Figure S2 — The homology analysis of A. capra based on gltA (A), groEL (B), and msp4 (C) sequences. [file Image_2.JPEG]
